# Adaptive lateral constraint-driven POCS interpolation method

**DOI:** 10.1038/s41598-026-39281-1

**Published:** 2026-03-01

**Authors:** Ziyu Qin, Shulin Pan, Jingyi Chen, Wenhua Wang, Yaojie Chen

**Affiliations:** 1 School of Computer Engineering, Chengdu Technological University, Chengdu, 611730 China; 2 School of Geoscience and Technology, Southwest Petroleum University, Chengdu, 610500 China; 3 School of Petroleum Engineering, The University of Tulsa, Tulsa, OK 74104 USA; 4 School of Computer Science, Chengdu Normal University, Chengdu, 611130 China

**Keywords:** Seismic data interpolation, Projection onto convex sets (POCS), Lateral constrained convex set, Convergence verification, Engineering, Mathematics and computing, Solid Earth sciences

## Abstract

The Projection onto Convex Sets (POCS) interpolation algorithm is widely adopted in seismic data processing, benefiting from its low computational complexity and strong data adaptability. However, the conventional POCS method fails to fully explore the inter-trace correlation information of seismic data, which leads to interpolation results with poor lateral continuity and high interpolation noise. To address this issue, this paper takes the traditional alternating projection framework for biconvex sets as the foundation, introduces a laterally constrained convex set, and thus effectively improves the interpolation quality of seismic data in terms of lateral continuity, signal-to-noise ratio (SNR) and interpolation accuracy. The specific research work is outlined as follows: First, the triple convex sets are defined in detail, the projection formula of the lateral constrained set is derived, and the triple convex set interpolation workflow is established. Second, the convergence of the new algorithm is theoretically proven, and its computational efficiency is compared and analyzed, which provides a reliable theoretical foundation for the stability of the algorithm. Finally, to verify the effectiveness of the proposed method, experiments are conducted on both synthetic seismic data and field seismic data, with a quantitative comparison of the interpolation accuracy between the two algorithms. The results demonstrate that the proposed algorithm significantly enhances interpolation accuracy while ensuring reconstruction efficiency, and therefore possesses excellent practical value.

**Supplementary Information:**

The online version contains supplementary material available at 10.1038/s41598-026-39281-1.

## Introduction

Seismic data acquisition is often constrained by economic, environmental, and technical conditions, resulting in incomplete data with gaps^1–3^. These missing data can affect subsequent processing steps such as migration and inversion, thereby reducing the accuracy of subsurface structural interpretation^4–10^. To address this issue, a variety of data reconstruction methods have been proposed successively. Based on core principles and technical approaches, seismic data interpolation methods can be categorized into traditional mathematical methods and machine learning methods.

Traditional methods are based on classical mathematical transformations and statistical laws as their core foundation, and achieve interpolation by directly exploring the intrinsic properties of data. Specifically, the predictive filtering method infers missing content by constructing a data autocorrelation prediction operator and leveraging the correlation information of adjacent traces^11–13^; methods based on the wave equation follow the physical laws of seismic wave propagation and complete spatial complementation of data by relying on technical means such as wavefield continuation^14– 15^; the wavefront attribute method relies on the kinematic characteristics of wavefronts (e.g., local traveltime parameters) to conduct targeted reconstruction of missing data^16– 17^; the POCS method is based on convex set geometry theory and gradually approaches the optimal solution through alternating projections between the data constraint set and the sparse constraint set^3,18–20^; regularization methods convert the interpolation problem into an optimization problem by introducing constraint conditions. For instance, the sparse transform method reconstructs signals by utilizing the sparsity of data in a specific transform domain and combining it with regularization constraints^21–28^; the rank reduction method achieves effective recovery of missing information based on the low-rank property of the data matrix^29– 30^.

With the rapid development of artificial intelligence technology, machine learning methods have become a research focus in the field of seismic data interpolation, thanks to their strong capabilities in nonlinear mapping learning and complex pattern extraction. Centered on data-driven approaches, these methods construct deep learning models to learn the mapping relationship between missing patterns and complete data from a large amount of labeled data, thereby achieving intelligent completion of missing trace sets^31–39^. However, machine learning methods have obvious limitations: on the one hand, they rely on large-scale high-quality labeled data; on the other hand, they incur high computational costs for model training and inference, and exhibit poor adaptability to unseen missing patterns.

Among the various methods mentioned above, the POCS method has been widely used in the field of seismic data interpolation due to its strong adaptability and stable performance^3,18–20^. In terms of its principle, the traditional POCS method mainly relies on the alternating projection of the data constraint set and the sparse constraint set to gradually approach the optimal solution. Although it can achieve basic data completion, it fails to fully explore the lateral correlation information of seismic data. In fact, the inter-trace similarity of seismic data stems from the spatial continuity of seismic wave propagation, and the event axes recorded by adjacent receiver traces have an inherent correlation in terms of traveltime and amplitude trends. The idea based on inter-trace similarity of seismic data has been widely applied in the field of seismic exploration and is often used as a key constraint condition^40–44^. However, the traditional POCS method ignores this key property, which not only limits the improvement of interpolation accuracy but also tends to introduce unnecessary interpolation noise during the data reconstruction process.

Based on this, this paper constructs a lateral constraint set guided by inter-trace correlation, which together with the traditional data constraint set and sparse constraint set forms a triple constraint system. This system can not only ensure the fidelity of interpolation results and the rationality in the frequency domain through the data constraint and sparse constraint, but also make full use of inter-trace correlation information by virtue of the lateral constraint, effectively avoiding the problems of noise introduction and structural distortion. Both theoretical derivation and experiments on actual seismic data show that the interpolation effect of the proposed algorithm is significantly better than that of the traditional POCS method.

The subsequent content of this paper is structured as follows: the “Methods” section defines the three constraint sets, expounds the alternating projection process of the triple convex sets, and proves the convergence of the algorithm based on convex analysis; the “Results” section takes synthetic pre-stack data and actual post-stack data as research objects, and compares the interpolation effects of the proposed algorithm and the traditional POCS method by combining the relative error (RE), peak signal-to-noise ratio (PSNR), and structural similarity index measure (SSIM); the “Conclusion” section summarizes the innovations and advantages of the proposed method, and puts forward ideas for future research.

## Methods

### Problem modeling and convex set definition

The essence of seismic data interpolation is to reconstruct complete data $z \in {{\mathbb{R}}^{{n_1} \times {n_2}}}$ from incomplete observed data $y \in {{\mathbb{R}}^{{n_1} \times {n_2}}}$, where ${n_1}$ denotes the number of time sampling points and ${n_2}$ denotes the number of seismic traces. With the help of POCS theory, signals that satisfy multiple constraint conditions can be alternately projected onto the convex sets corresponding to each constraint, gradually approaching the optimal solution for interpolation. On the basis of the conventional POCS interpolation algorithm, this paper further introduces a horizontal constraint and constructs three types of convex sets to improve the effect of seismic data interpolation.

#### Data constraint set A

Data constraint set A consists of all data that satisfy the condition “the original observed data remain unchanged at the positions of known data”, which is intended to ensure the data fidelity of interpolation results. Its mathematical expression is as follows:

1 $$A=\left\{ {\mu |M \odot \mu =M \odot y} \right\}$$

where $M \in {{\mathbb{R}}^{{n_1} \times {n_2}}}$ represents the sampled mask matrix, where 0 corresponds to missing data and 1 corresponds to existing data; $\odot$ denotes element-wise multiplication. y is the observed data, and $\mu$ is the result after projection onto other convex sets.

#### Sparse constraint set B

Sparse constraint set B in the frequency domain includes all signals that satisfy the sparsity condition in the frequency domain. It leverages the energy concentration characteristic of seismic data in the frequency domain to suppress artifact signals during the interpolation process, and is defined as follows:

2 $$B=\left\{ {x|{{\left\| {\Gamma \left( x \right) \odot {\chi _{\left| {\Gamma \left( x \right)} \right| \geqslant \tau }}} \right\|}_0} \leqslant \delta } \right\}$$

here, $x \in {{\mathbb{R}}^{{n_1} \times {n_2}}}$ denotes the input data, $\Gamma \left( x \right)$ denotes the sparse transform, ${\chi _{\left| {\Gamma \left( x \right)} \right| \geqslant \tau }}$ is the indicator function (taking the value 1 when $\left| {\Gamma \left( x \right)} \right| \geqslant \tau$ is satisfied, and 0 otherwise), $\tau$ represents the frequency-domain threshold, and $\delta$ is the preset sparsity. Although the ${L_0}$ norm itself is non-convex, the set B can satisfy the definition of a convex set through screening with the fixed-threshold indicator function, which ensures the effectiveness of the projection operation.

#### Lateral constraint set C

Lateral constraint set C is constructed based on the inter-trace similarity of seismic data. It can quantify inter-trace differences using a second-order difference operator and enforce the interpolation results to maintain lateral continuity. It is defined as follows:

3 $$C=\left\{ {x|{{\left\| {xD} \right\|}_1} \leqslant \gamma } \right\}$$

where $D \in {{\mathbb{R}}^{{n_2} \times {n_2}}}$ denotes the second-order difference matrix, and its elements satisfy the following condition:

4 $$D\left( {j,i} \right)=\left\{ {\begin{array}{*{20}{c}} { - 1,{\text{ }}j=i - 1} \\ {2,{\text{ }}j=i} \\ { - 1,j=i+1} \\ {0,other} \end{array}} \right.$$

The first trace $i=1$ and the last trace $i={n_2}$ are corrected using the one-sided second-order difference ($D\left( {1,1} \right)=1,{\text{ }}D\left( {2,1} \right)= - 1$, $D\left( {{n_2} - 1,{n_2}} \right)=1,{\text{ }}D\left( {{n_2},{n_2}} \right)= - 1$); $\gamma$ is the inter-trace difference threshold; ${\left\| \cdot \right\|_1}$ is the ${L_1}$ norm, which is used to enhance the ability to suppress inter-trace abrupt signals.

### Definition of projection operator

The core function of a projection operator is to map any input data to the closest point within a convex set. For the three types of convex sets mentioned above, the projection operators are defined as follows:

#### Projection operator for the data constraint set ${P_A}$

${P_A}$ is used to project the input data x onto the convex set A. It preserves the information of known data and retains the current estimated values at missing positions. Its mathematical expression is as follows:

5 $${P_A}\left( x \right)=M \odot y+\left( {I - M} \right) \odot x$$

where, $I \in {{\mathbb{R}}^{{n_1} \times {n_2}}}$denotes the identity matrix; $M \odot y$ensures that the data at known positions remain unchanged, while $\left( {I - M} \right) \odot x$ retains the current iterative results at missing positions. This achieves a balance between preserving the fidelity of known data and updating the missing data.

#### Projection operator for the frequency-domain sparse set ${P_B}$

${P_B}$implements the sparse constraint through a frequency-domain thresholding operation. It projects the input data x onto the convex set B, retaining only the components whose frequency-domain amplitude is higher than the threshold $\tau$. Its expression is as follows:

6 $${P_B}\left( x \right)={\Gamma ^{ - 1}}\left( {\Gamma \left( x \right) \odot {\chi _{\left| {\left( {\Gamma \left( v \right)} \right)} \right| \geqslant \tau }}} \right)$$

where $\Gamma \left( \cdot \right)$ denotes the sparse transform, ${\Gamma ^{ - 1}}\left( \cdot \right)$ denotes the inverse transform. $\tau$ is dynamically updated using an exponential decay strategy^19^:

7 $${\tau _k}={\tau _{\hbox{max} }}\exp \left( { - \frac{{\ln \left( {{\tau _{\hbox{max} }}/{\tau _{\hbox{min} }}} \right)}}{{N - 1}}k} \right)$$

where ${\tau _{\hbox{max} }}$ is the initial threshold, ${\tau _{\hbox{min} }}={10^{ - 4}}{\tau _{\hbox{max} }}$,*k* denotes the number of iterations $\left( {0 \leqslant k \leqslant N - 1} \right)$,* N*denotes the maximum number of iterations.

#### Projection operator for the lateral constraint set ${P_C}$

**Proposition:**

${P_C}$minimizes the inter-trace difference energy via gradient descent and projects the input data x onto the convex set C. Its expression is as follows:

8 $${P_C}\left( x \right)=x - \lambda sign\left( {xD} \right) \cdot {D^T}$$

where, $\lambda$denotes the Lagrange multiplier, which is used to balance the lateral constraint and the fidelity term; ${D^T}$ denotes the transpose of the second-order difference matrix; and $sign\left( \cdot \right)$ denotes the sign function.

**Proof of proposition:**

The core of projecting onto the convex set C is to map the input data x to the closest point within the convex set C. The goal is to minimize the difference between the projection result and the input data while satisfying the lateral continuity constraint. Essentially, the projection operator ${P_C}\left( x \right)$ solves the following optimization problem:

9 $${P_C}\left( x \right)=\arg {\hbox{min} _{z \in C}}\frac{1}{2}\left\| {z - x} \right\|_{2}^{2}$$

where $z \in {{\mathbb{R}}^{{n_1} \times {n_2}}}$denotes the projected data, $\left\| \cdot \right\|_{2}^{{}}$denotes the $L_{2}^{{}}$ norm. By introducing the Lagrange multiplier $\lambda \geqslant 0$, a Lagrangian function is constructed to convert the constrained optimization problem into an unconstrained one:

10 $$L\left( {x,\lambda } \right)=\frac{1}{2}\left\| {z - x} \right\|_{2}^{2}+\lambda \left( {{{\left\| {xD} \right\|}_1} - \gamma } \right)$$

Since the ${L_1}$ norm is not differentiable at zero, the subgradient is used instead of the conventional gradient to solve for the extremum.

Taking the subgradient of $L\left( {x,\lambda } \right)$ with respect to x can be decomposed into two separate calculations:

(1)The gradient of the ${L_2}$ norm term $\frac{1}{2}\left\| {z - x} \right\|_{2}^{2}$ is $z - x$;

(2)According to convex analysis theory, for the ${L_1}$ norm term ${\left\| {AB} \right\|_1}$ in matrix form (where* A* is the variable matrix and* B* is the fixed matrix), its subgradient is $sign\left( {AB} \right) \cdot {B^T}$.$sign\left( \cdot \right)$ denotes the sign function

11 $$sign\left( a \right)=\left\{ {\begin{array}{*{20}{c}} {1,a>0} \\ { - 1,a<0} \\ {[ - 1,1],a=0} \end{array}} \right.$$

Set the subgradient of the Lagrangian function to zero:

12 $$z - x+\lambda sign\left( {xD} \right) \cdot {D^T}=0$$

After rearrangement, we obtain:

13 $$z=x - \lambda sign\left( {xD} \right) \cdot {D^T}$$

For the Lagrange multiplier $\lambda$ in Eq. (8), which balances lateral constraint strength and interpolation result fidelity, its rationality directly determines the quality of interpolation performance. When λ is excessively large, the lateral constraint dominates, which will over-smooth the details of seismic signals and even distort natural lateral difference features such as faults and lithological interfaces, leading to abnormal deviations between some traces and the original data; when $\lambda$ is excessively small, the lateral constraint is nearly ineffective, and the interpolation effect degrades to the level of the traditional POCS algorithm, making it difficult to solve the core problems of poor lateral continuity and high interpolation noise.

Based on systematic experimental verification with a large amount of synthetic data and actual seismic data, the optimal value range of $\lambda$ lies in the order of 10⁻³-10⁻¹, among which 0.008–0.12 is the core effective interval. Within this interval, the optimal balance between lateral continuity and data fidelity can be achieved.

In practical applications, the “stepwise testing method” can be adopted to determine the optimal λ for specific data. For example, select discrete values (such as 0.008, 0.02, 0.06, 0.08, 0.12) within the range of 0.008 ~ 0.12, calculate the corresponding evaluation indicators for each value, and select the value with the optimal comprehensive indicators.

#### Adaptive projection operator ${P_C}$ for lateral constraint

Using the lateral difference operator can constrain interpolation by leveraging inter-trace correlation. However, when there is a large difference between two adjacent traces, the interpolation result will tend to approach the adjacent trace and deviate from the true value—even performing worse than the conventional POCS algorithm. Therefore, to better adapt to lateral variations, a similarity matrix is constructed to guide the difference based on the projection operator ${P_C}$, with the steps as follows:

1. Divide each seismic trace into time windows $W=\left\{ {\left( {{s_k},{e_k}} \right)|k=1,2, \cdots ,K} \right\}$ along the time direction with length* L*, where ${s_k}=\left( {k - 1} \right)L+1$ is the starting sampling point of the k-th time window, and ${e_k}=kL$ is the ending sampling point (if the last time window has fewer than * L* sampling points, take ${e_k}={n_1}$, and* K* is the total number of time windows). The value of L is determined by the number of sampling points covering 1–2 dominant frequency periods of the seismic data, which is calculated as $L=\left( {1/{f_m} \times 1000} \right)/s \times k$ (where ${f_m}$ represents the dominant frequency in Hz, s is the time sampling rate, k is the period coefficient, an empirical value ranging from 1 to 2. For the processing of large-volume seismic data, the value of k can be appropriately increased to expand the time window and further improve the computational efficiency.
2. Let the set of known seismic traces be* J*. For any target trace $j\left( {1 \leqslant j \leqslant {n_2}} \right)$, calculate the distance $dist=\left| {j - j^{\prime}} \right|\left( {j^{\prime} \in J} \right)$ between target trace * j* and each trace in* J*, and select the two known traces ${j_1}^{\prime },{j_2}^{\prime }$ with the smallest distances.

For each time window $\left( {{s_k},{e_k}} \right)$, calculate the correlation coefficient between target trace* j* and known traces ${j_1}^{\prime },{j_2}^{\prime }$ within this time window. Let the data within the time window be ${x_{{s_k}:{e_{k,}}j}},{x_{{s_k}:{e_{k,}}{{j^{\prime}}_1}}},{x_{{s_k}:{e_{k,}}{{j^{\prime}}_2}}}$ respectively, then the similarity $sim\left( {k,j} \right)$ is:

14 $$sim\left( {k,j} \right)=\frac{1}{2}\left[ {\frac{{conv\left( {{x_{{s_k}:{e_{k,}}j}},{x_{{s_k}:{e_{k,}}{{j^{\prime}}_1}}}} \right)}}{{\sigma \left( {{x_{{s_k}:{e_{k,}}j}}} \right)\sigma \left( {{x_{{s_k}:{e_{k,}}{{j^{\prime}}_1}}}} \right)}}+\frac{{conv\left( {{x_{{s_k}:{e_{k,}}j}},{x_{{s_k}:{e_{k,}}{{j^{\prime}}_2}}}} \right)}}{{\sigma \left( {{x_{{s_k}:{e_{k,}}j}}} \right)\sigma \left( {{x_{{s_k}:{e_{k,}}{{j^{\prime}}_2}}}} \right)}}} \right]$$

among them, $conv\left( { \cdot , \cdot } \right)$ denotes the covariance, and $\sigma \left( { \cdot , \cdot } \right)$ denotes the standard deviation. If the standard deviation is less than ${10^{ - 10}}$, set $sim\left( {k,j} \right)=0$. Assign the similarity $sim\left( {k,j} \right)$ of time window $\left( {{s_k},{e_k}} \right)$ to all sampling points within this time window to form a similarity matrix $sim \in {{\mathbb{R}}^{{n_1} \times {n_2}}}$ with the same dimension as the original data. In actual data, the similarity of random noise is usually 0. Therefore, after obtaining the similarity matrix, the elements that originally have a similarity of 0 need to be set to 1 to enable the difference operator to suppress random noise.

(3) Construct a neighboring trace constraint matrix ${x_c} \in {{\mathbb{R}}^{{n_1} \times {n_2}}}$. For a time window $\left( {{s_k},{e_k}} \right)$ of seismic trace* j*, if $sim\left( {k,j} \right)<\zeta$, store the average value of the seismic data at the corresponding position of adjacent traces in the corresponding position of ${x_c}$; otherwise, store the data of the current trace in the corresponding time window:

15 $${x_c}\left( {{s_k},{e_k},j} \right)=\left\{ {\begin{array}{*{20}{c}} \begin{gathered} \frac{1}{2}\left( {x\left( {{s_k},{e_k},j - 1} \right)+x\left( {{s_k},{e_k},j+1} \right)} \right),sim\left( {k,j} \right) \geqslant \zeta ,1<j<{n_2} \hfill \\ x\left( {{s_k},{e_k},j+1} \right){\text{ or }}x\left( {{s_k},{e_k},j - 1} \right),sim\left( {k,j} \right) \geqslant \zeta ,j=1{\text{ or }}j={n_2} \hfill \\ \end{gathered} \\ {x\left( {{s_k},{e_k},j} \right),sim\left( {k,j} \right)<\zeta } \end{array}{\text{ }}} \right.$$

among them, $\zeta \in \left[ {0,1} \right]$ is the similarity threshold, determined by data characteristics.

(4) Replace $xD$ with the difference between the input data x and the neighboring trace constraint matrix ${x_c}$ to realize the Adaptive Lateral Constraint POCS algorithm (ALC-POCS):

16 $$z=x - \lambda sign\left( {x - {x_c}} \right) \cdot {D^T}$$

### Iteration process of ALC-POCS

Based on the above projection operator, a triple convex set alternating projection process with data constraint, frequency-domain sparsity, and adaptive lateral constraint is constructed, with the specific steps as Algorithm 1.

**Algorithm 1:**
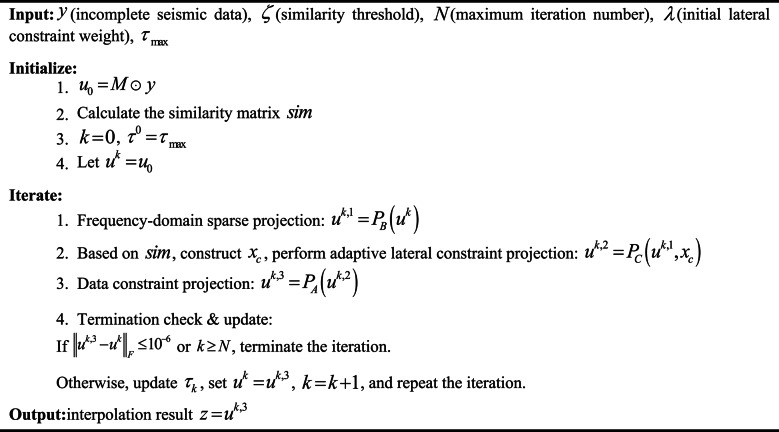
ALC-POCS.

**Fig. 1:**
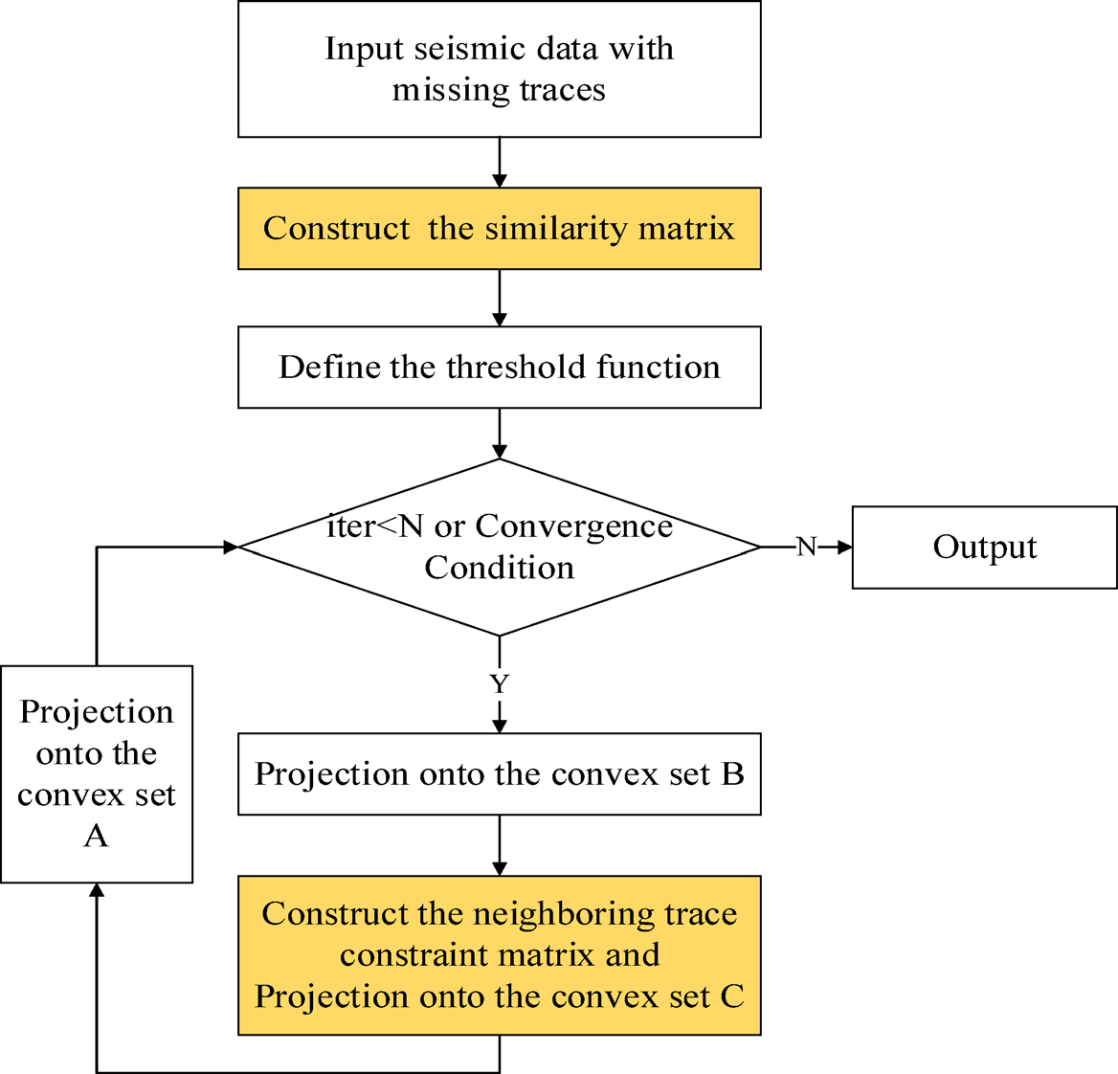
Calculation flowchart of ALC-POCS.

Figure 1 shows the calculation flowchart of the ALC-POCS algorithm. Compared with the traditional POCS interpolation, an adaptive lateral constraint convex set C (the yellow part) is added. This subsection only elaborates on the relevant definitions and core process of the ALC-POCS algorithm. Based on the core premises of the non-emptiness of the intersection of convex sets and the nonexpansiveness of projection operators, the iterative convergence of the proposed algorithm can be strictly proven via the classical POCS convergence theorem, with detailed derivation processes provided in Appendix A.

### Computational efficiency comparison with AK-POCS algorithm

The proposed ALC-POCS algorithm and the baseline AK-POCS algorithm both achieve seismic data interpolation based on an iterative projection framework. Their asymptotic time complexity orders are consistent, but the improved version introduces additional linear computational overhead due to dynamic trace-wise constraints and time-window similarity calculations. A detailed comparison of their total time complexity is provided below:

The AK-POCS algorithm has no preprocessing stage, with the iterative process dominated by the frequency-domain FFT projection as the core computational bottleneck. Its total time complexity is $O\left( {T \cdot MN\log \left( {MN} \right)} \right)$, where T represents the actual convergence iterations, and M and N denote the number of time samples and traces in the seismic data, respectively. The 2D FFT operation, with complexity $O\left( {T \cdot MN\log \left( {MN} \right)} \right)$, dominates the overall computational load, while linear operations such as data-domain projection account for a negligible proportion.

The ALC-POCS algorithm builds on the baseline framework by adding modules for non-overlapping time-window partitioning, trace-wise similarity calculation, and dynamic constraint matrix construction.Its total time complexity is $O\left( {MN+T \cdot \left( {MN\log \left( {MN} \right)+MN} \right)} \right)$. Here, $O\left( {MN} \right)$ represents the one-time computational overhead of the pre-iteration preprocessing stage; during iteration, in addition to retaining the core frequency-domain FFT projection ($O\left( {MN\log \left( {MN} \right)} \right)$), an additional $O\left( {MN} \right)$ linear computation is introduced for dynamic constraint matrix construction.

Both algorithms take the 2D FFT operation as their core computational bottleneck, so their asymptotic time complexity orders are consistent and belong to polynomial complexity.The additional overhead of the improved version only manifests as an increase in the coefficient of the linear term ($O\left( {MN} \right)$), without altering the overall complexity growth trend of the algorithm.This confirms that the improved version achieves enhanced interpolation accuracy and robustness at the cost of a slight reduction in computational efficiency, representing a reasonable trade-off between algorithm performance and interpolation results.

## Results

To systematically verify the effectiveness, noise resistance, and engineering applicability of the ALC-POCS interpolation method proposed in this paper, we selected a single-shot record corresponding to the Marmousi model and post-stack seismic data from an actual work area for testing, respectively. Both types of data were evaluated qualitatively and quantitatively against the traditional AK-POCS method^18^. The experimental parameters were set uniformly: the maximum number of iterations $N=100$, and F-K transform was adopted as the sparse transform to ensure consistent experimental conditions.

### Example with synthetic data

**Fig. 2:**
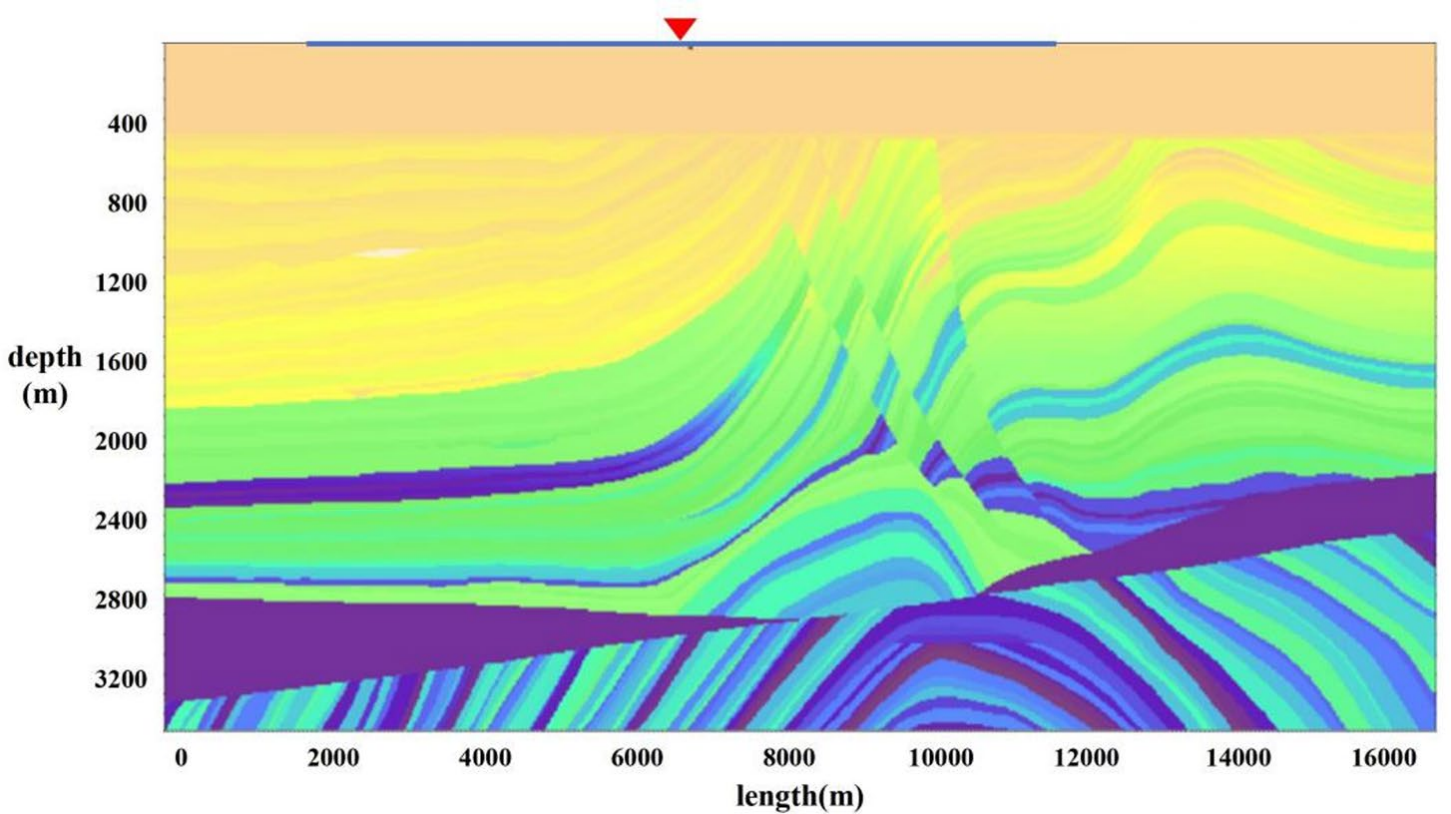
Marmousi model. The red triangles denote shot points, and the blue lines represent the receiver coverage range.

**Fig. 3:**
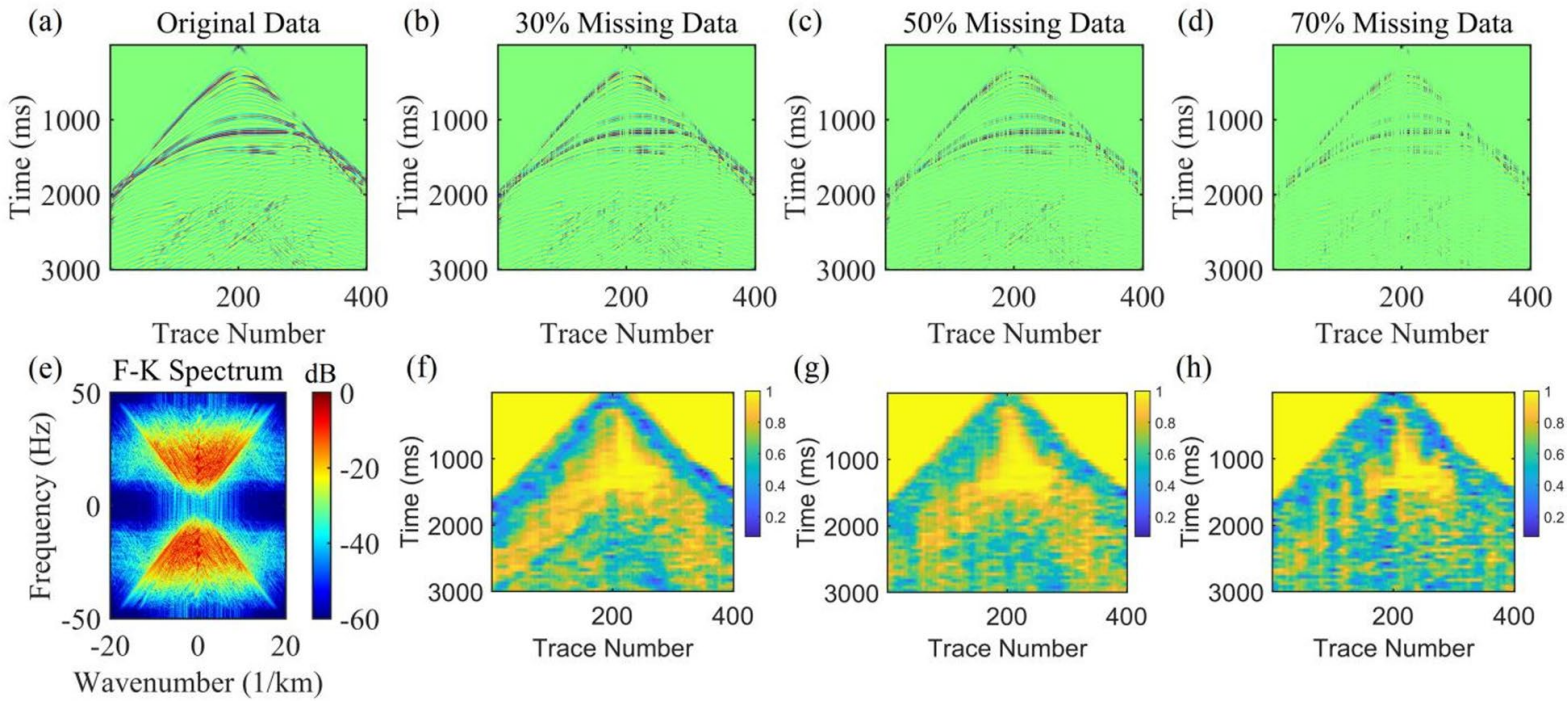
Single-shot seismic gather and correlation matrices derived from forward modeling based on the Marmousi model. (**a**) shows the original single-shot gather without missing traces, (**b**)-(**d**) denote the gather data with 30%, 50% and 70% randomly missing traces in sequence, (**e**) is the F-K spectrum of the original data, and (**f**)-(**h**) correspond to the data correlation matrices under the aforementioned missing ratios, respectively.

The Marmousi model covers complex fault-block structures, lithological interface abrupt change zones, and gentle formations (Fig. 2). For the single-shot record of this model, the time sampling rate is set to 1 ms with a total time length of 3000 ms. Each shot is equipped with 400 receivers at a spacing of 25 m, and the maximum offset reaches ± 5000 m. This record has event characteristics of local continuity and global complexity, which can effectively test the algorithm’s ability to model inter-trace correlation and its noise resistance performance. Figures 3(a)-(d) show the original single-shot record, as well as seismic records with 30%, 50%, and 70% random missing data. Figures 3(f)-(h) present the calculation results of the seismic data correlation matrix under different missing ratios. A higher correlation value indicates a greater probability of applying lateral constraints. It can be clearly observed from the figure that as the data missing ratio increases, the yellow areas in the correlation matrix gradually decrease. Moreover, the distribution of yellow areas is consistent with cognitive rules. The inclined areas have poor correlation, corresponding to a lower probability of applying lateral constraints; the horizontal event areas have good correlation, corresponding to a higher probability of applying lateral constraints; the random noise areas are overall yellow, indicating that noise suppression in these areas can be achieved through lateral constraints. Figure 3(e) shows the F-K spectrum of the original seismic record, providing a key reference benchmark for verifying the validity of subsequent interpolation results. This spectrum was plotted by normalizing the amplitude spectrum derived from the 2D Fourier transform of the original seismic record, followed by conversion to the decibel scale.

**Fig. 4:**
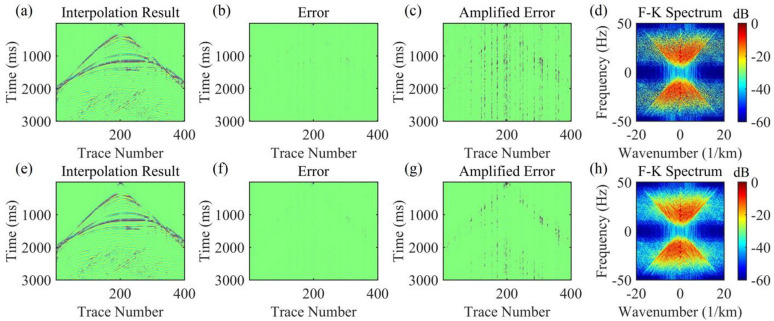
Interpolation results of Marmousi model prestack gather with 30% random missing data. (**a**)-(**d**) Interpolation result, error, amplified error, and F-K spectrum of the AK-POCS method; (**e**)-(**h**) Interpolation result, error, amplified error, and F-K spectrum of the method proposed in this paper.

**Fig. 5:**
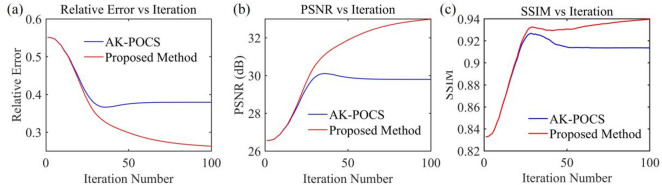
Plots of (**a**) RE, (**b**) PSNR, and (**c**) SSIM versus number of iterations for prestack gather with 30% missing data.

For the data to be interpolated with 30% randomly missing traces, the AK-POCS algorithm and ALC-POCS algorithm were used for interpolation respectively. The results show that both algorithms can restore the continuous morphology of events after interpolation (Figs. 4a, e), with small actual errors (Figs. 4b, f). After amplifying the error amplitude, it can be found that the interpolation error of the AK-POCS algorithm is larger (Figs. 4c, g). From the F-K spectra of the interpolation results of the two algorithms (Figs. 4d, h), the AK-POCS algorithm introduces a large amount of random noise in the F-K spectrum, which differs more from the F-K spectrum of the original data (Fig. 3e). Figure 5 shows the variation trends of different indicators of the AK-POCS algorithm and ALC-POCS algorithm with the number of iterations. The RE, PSNR, and SSIM of the ALC-POCS algorithm are all better than those of the AK-POCS algorithm.

**Fig. 6:**
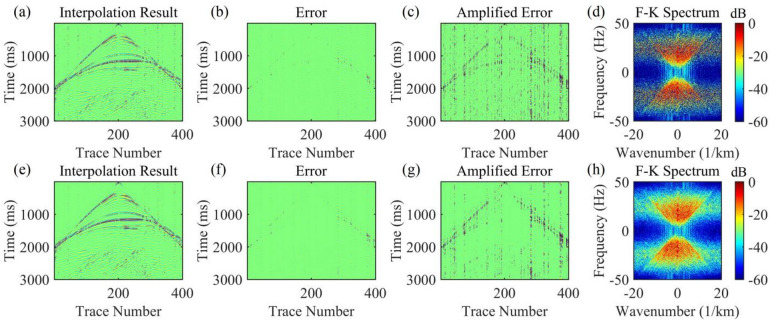
Interpolation results of Marmousi model prestack gather with 50% random missing data. (**a**)-(**d**) Interpolation result, error, amplified error, and F-K spectrum of the AK-POCS method; (**e**)-(**h**) Interpolation result, error, amplified error, and F-K spectrum of the method proposed in this paper.

**Fig. 7:**
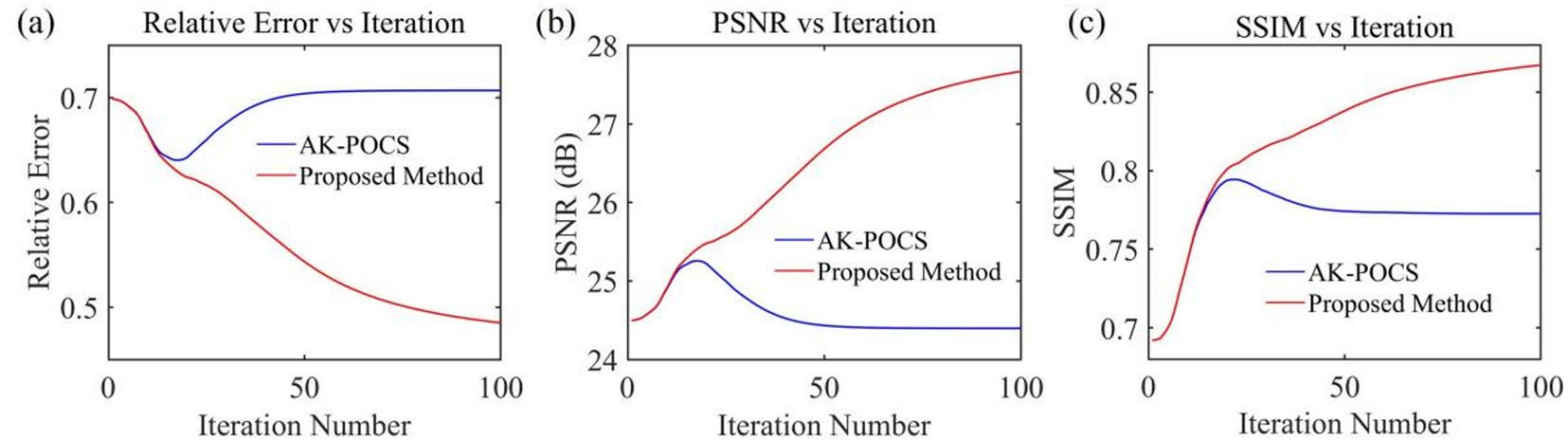
Plots of (**a**) RE, (**b**) PSNR, and (**c**) SSIM versus number of iterations for prestack gather with 50% missing data.

Under the scenario of 50% random missing data, the seismic events are severely discretized, with a maximum of 8 consecutive missing traces (Fig. 3(c)). AK-POCS exhibits unsatisfactory interpolation performance when facing multiple consecutive missing traces and introduce strong interpolation noise. In contrast, the method proposed in this paper significantly improves the interpolation effect through adaptive lateral constraints (Figs. 6c, g), and the SNR of the seismic records is higher than that of the AK-POCS algorithm (Figs. 6a, e). From the perspective of F-K spectra (Figs. 6d, h), the effective signals in the F-K spectrum of the AK-POCS algorithm are severely disturbed by noise, showing a greater difference from the F-K spectrum of the original data (Fig. 3e). The ALC-POCS algorithm outperforms the AK-POCS algorithm in RE, PSNR, and SSIM (Fig. 7).

**Fig. 8:**
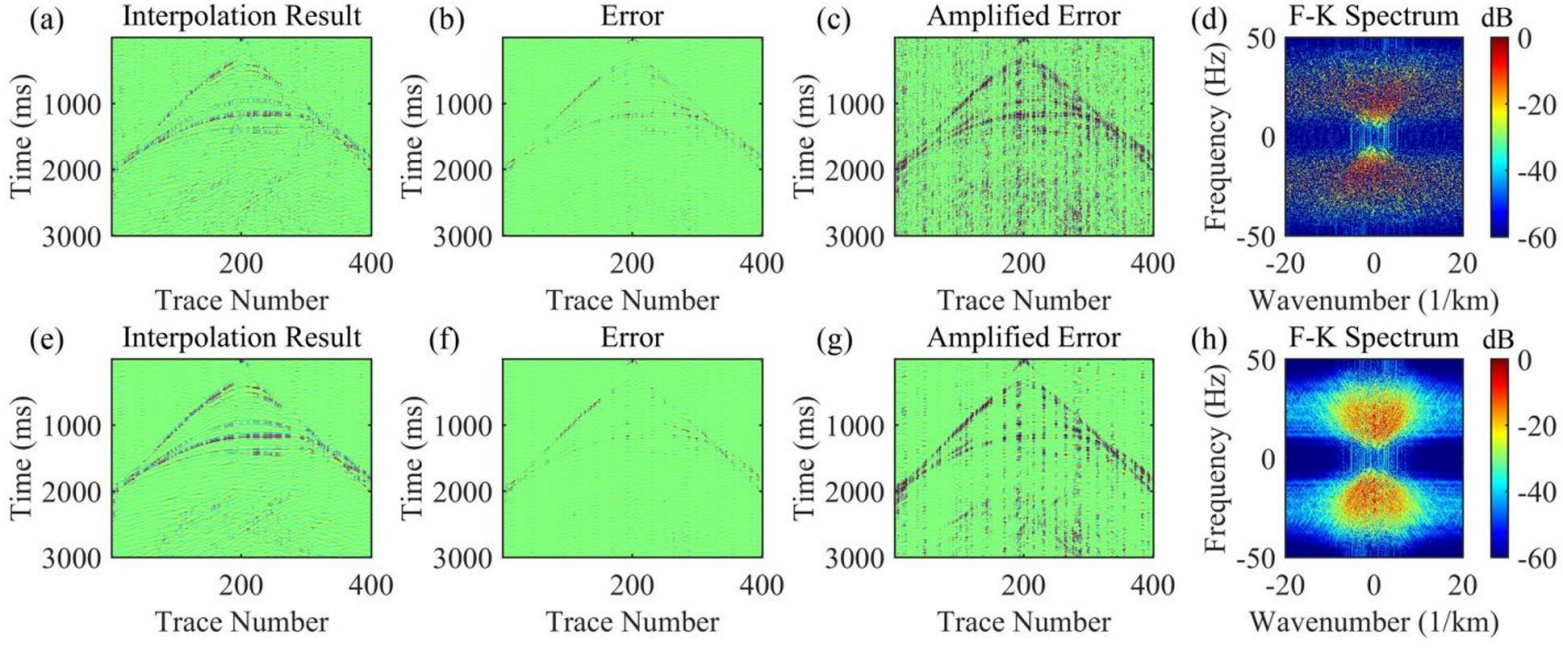
Interpolation results of Marmousi model prestack gather with 70% random missing data. (**a**)-(**d**) Interpolation result, error, amplified error, and F-K spectrum of the AK-POCS method; (**e**)-(**h**) Interpolation result, error, amplified error, and F-K spectrum of the method proposed in this paper.

**Fig. 9:**
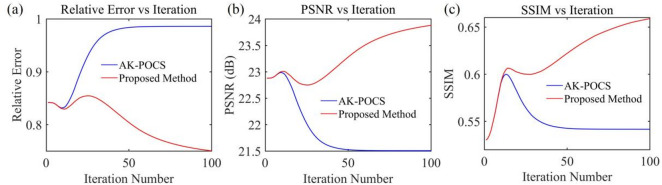
Plots of (**a**) RE, (**b**) PSNR, and (**c**) SSIM versus number of iterations for prestack gather with 70% missing data.

**Fig. 10:**
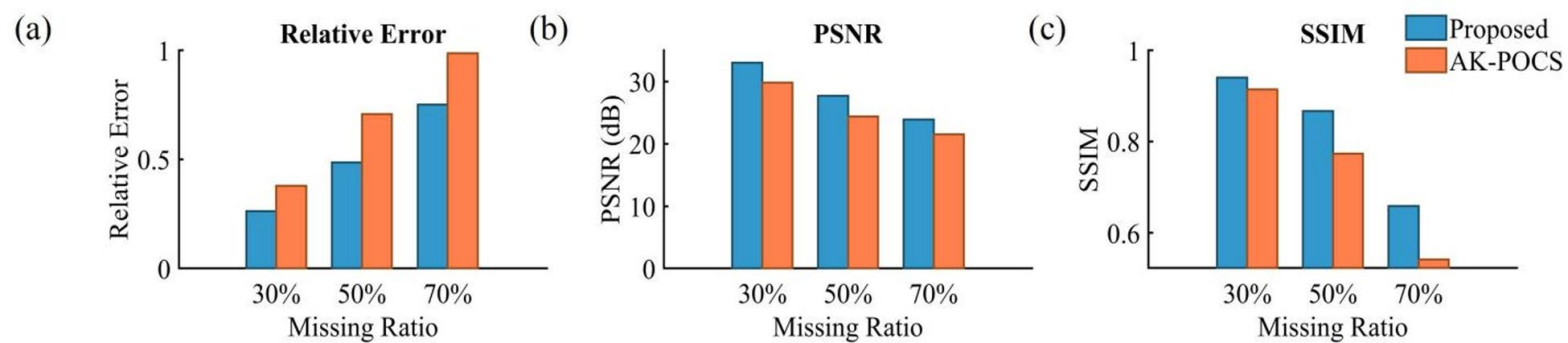
Comparison of indicators between the interpolation results of the AK-POCS algorithm and the proposed method with the expected data under different missing ratios: (**a**) RE; (**b**) PSNR; (**c**) SSIM.

The 70% random missing scenario is an extreme case, where only 30% of valid information is retained in the data to be interpolated. The seismic events are almost completely discretized, with a maximum of 15 consecutive missing traces. Both algorithms yield unsatisfactory reconstruction results (Figs. 8a, e). However, compared with the AK-POCS algorithm (Fig. 8c), the error data of the ALC-POCS algorithm contains fewer effective signals (Fig. 8g); the effective signals in the F-K spectrum of the AK-POCS algorithm are completely covered by noise, while the features of the ALC-POCS algorithm remain relatively clear (Figs. 8d, h). Figure 9 demonstrates that the ALC-POCS algorithm still outperforms the AK-POCS algorithm in indicators such as RE, PSNR, and SSIM even under high missing rates. Figure 10 compares the performance of the AK-POCS and ALC-POCS algorithms under different data missing rates, with the indicators between the interpolated data and the ground truth data as the evaluation metrics. The results show that the three indicators of both algorithms exhibit a downward trend as the missing rate increases; when the data missing rate reaches 70%, the performance metrics of the ALC-POCS algorithm are basically equivalent to those of the AK-POCS algorithm at a 50% missing rate. Overall, in geological scenarios such as complex fault blocks and abrupt lithological interfaces, the proposed method can more accurately restore the spatial morphology and continuity of event axes, effectively avoiding the common problems of event axis blurring or distortion in traditional algorithms, and achieving superior fidelity of geological features.

### Post-stack seismic records

Post-stack seismic data from an oil and gas exploration project in a basin in eastern China were selected for interpolation experiments and analysis. In the experiments, 300 seismic traces were extracted, and random missing treatments of 30%, 50% and 70% were applied to them respectively. The seismic data had a sampling rate of 1 millisecond, with each trace containing 1500 sampling points. The target data cover two typical regions with gradual and abrupt structural changes, which can effectively test the performance of the interpolation method proposed in this paper.

**Fig. 11:**
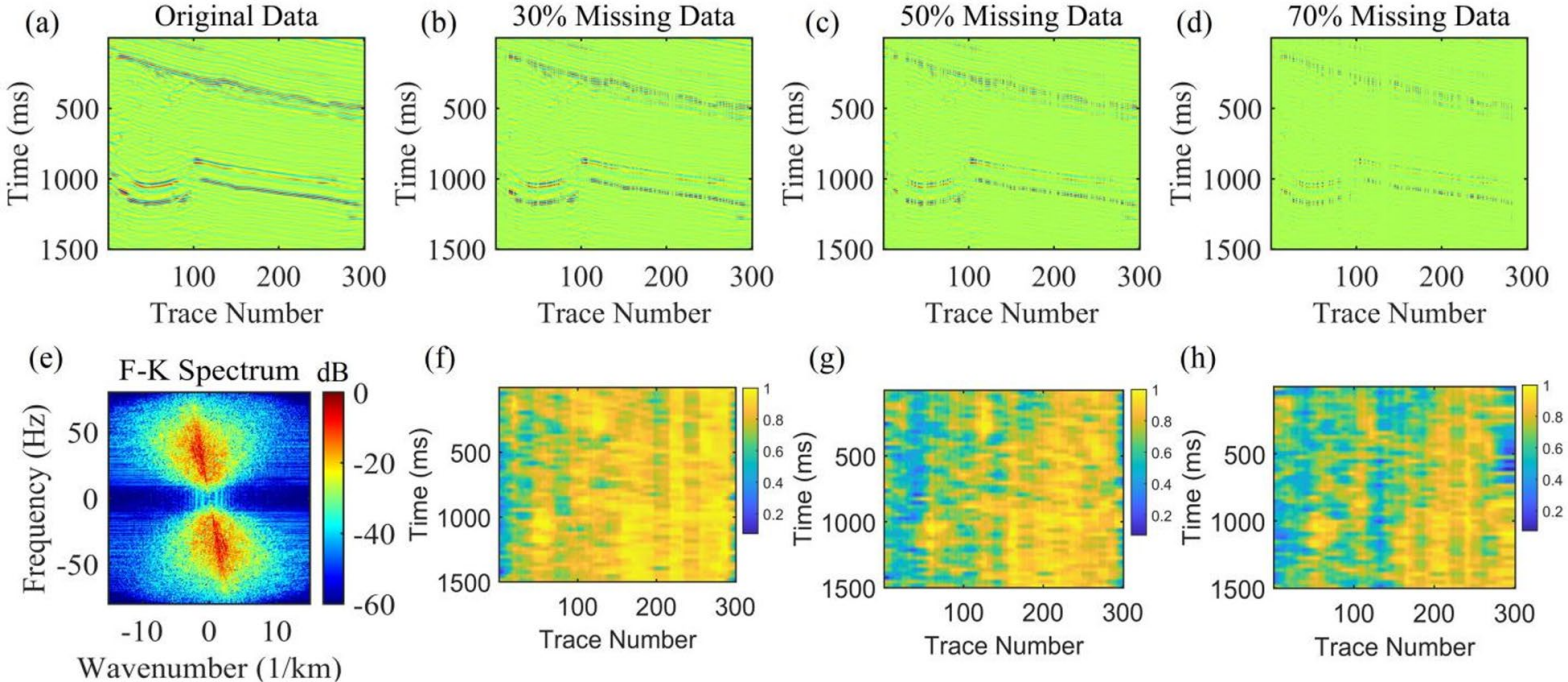
Actual post-stack seismic record. (**a**) shows the original single-shot gather without missing traces, (**b**)-(**d**) denote the gather data with 30%, 50% and 70% randomly missing traces in sequence, (**e**) is the F-K spectrum of the original data, and (**f**)-(**h**) correspond to the data correlation matrices under the aforementioned missing ratios, respectively.

Figure 11a shows the post-stack seismic records of an actual work area. The data overall changes gently, with certain local structural abrupt changes. Figures 11b-d present the seismic records with 30%, 50%, and 70% random missing data respectively. As the missing ratio increases, the continuity of seismic events is gradually destroyed. Figure 11f-h show the correlation matrices of seismic records under different data missing ratios. From the distribution characteristics of the matrices, it can be seen that the regions with high correlation coefficients (yellow) highly correspond to the geological units with gentle underground formation changes. In such regions, the seismic events have strong continuity and stable lateral information correlation, and the lateral constraint information of adjacent traces can be more fully utilized during interpolation to improve the geological authenticity of interpolation results. In contrast, the regions with low correlation coefficients (blue) are concentrated in two types of scenarios: one is the regions with abrupt formation lithology changes and complex structural forms (such as faults and lithological interfaces), where the seismic reflection characteristics themselves have large differences and weak lateral correlation; the other is the regions with high data missing ratios, where the information gaps caused by missing traces further weaken the correlation of adjacent traces. For regions with low correlation coefficients, traditional methods need to be switched to during interpolation to balance data rationality and computational stability, and avoid distortion of geological features caused by forced application of lateral constraints. Figure 11e shows the F-K spectrum of the post-stack seismic record.

**Fig. 12:**
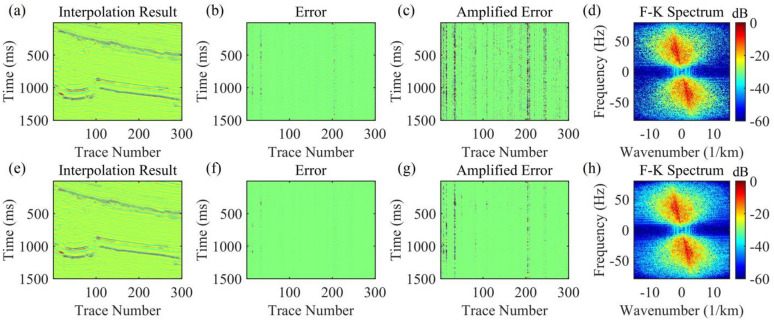
Interpolation results of the actual post-stack record with 30% missing data. (**a**)-(**d**) Interpolation result, error, amplified error, and F-K spectrum of the AK-POCS method; (**e**)-(**h**) Interpolation result, error, amplified error, and F-K spectrum of the method proposed in this paper.

Figure 12 shows the comparison of interpolation results for real post-stack records with 30% missing data. Figures 12a-d present the interpolation results of the AK-POCS method, although it restores seismic events to a certain extent, there are still missing details. Figures 12e-h show the interpolation results of the method proposed in this paper. From the amplified error amplitude plots, the proposed method (Fig. 12c) has smaller interpolation errors than the AK-POCS algorithm (Fig. 12g). Its F-K spectrum is highly consistent with the original spectrum (Fig. 11e) in terms of effective energy distribution and wavenumber continuity, accurately restoring the frequency-wavenumber domain information of the seismic wavefield. Both the interpolation accuracy and wavefield information restoration capability are significantly superior to those of the AK-POCS method.

**Fig. 13:**
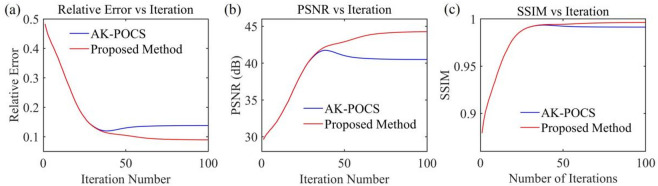
Plots of (**a**) RE, (**b**) PSNR, and (**c**) SSIM versus the number of iterations for post-stack seismic records with 30% missing data.

**Fig. 14:**
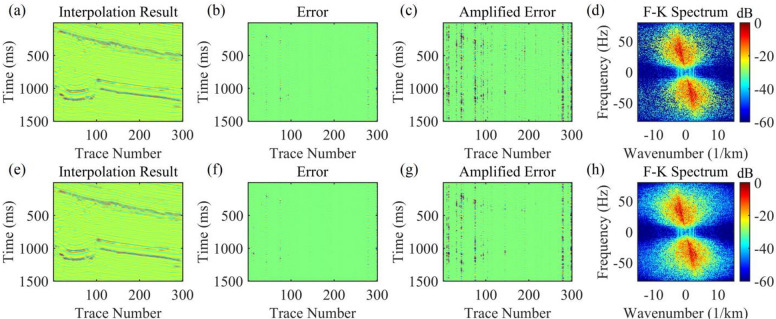
Interpolation results of the actual post-stack record with 50% missing data. (**a**)-(**d**) Interpolation result, error, amplified error, and F-K spectrum of the AK-POCS method; (**e**)-(**h**) Interpolation result, error, amplified error, and F-K spectrum of the method proposed in this paper.

**Fig. 15:**
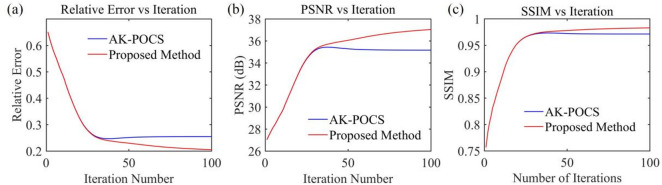
Plots of (**a**) RE, (**b**) PSNR, and (**c**) SSIM versus the number of iterations for post-stack seismic records with 50% missing data.

**Fig. 16:**
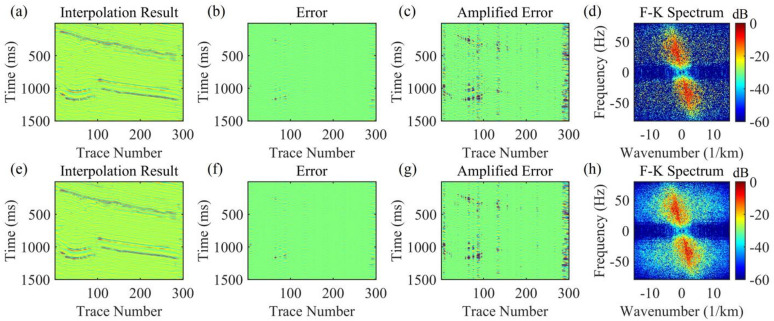
Interpolation results of the actual post-stack record with 70% missing data. (**a**)-(**d**) Interpolation result, error, amplified error, and F-K spectrum of the AK-POCS method; (**e**)-(**h**) Interpolation result, error, amplified error, and F-K spectrum of the method proposed in this paper.

**Fig. 17:**
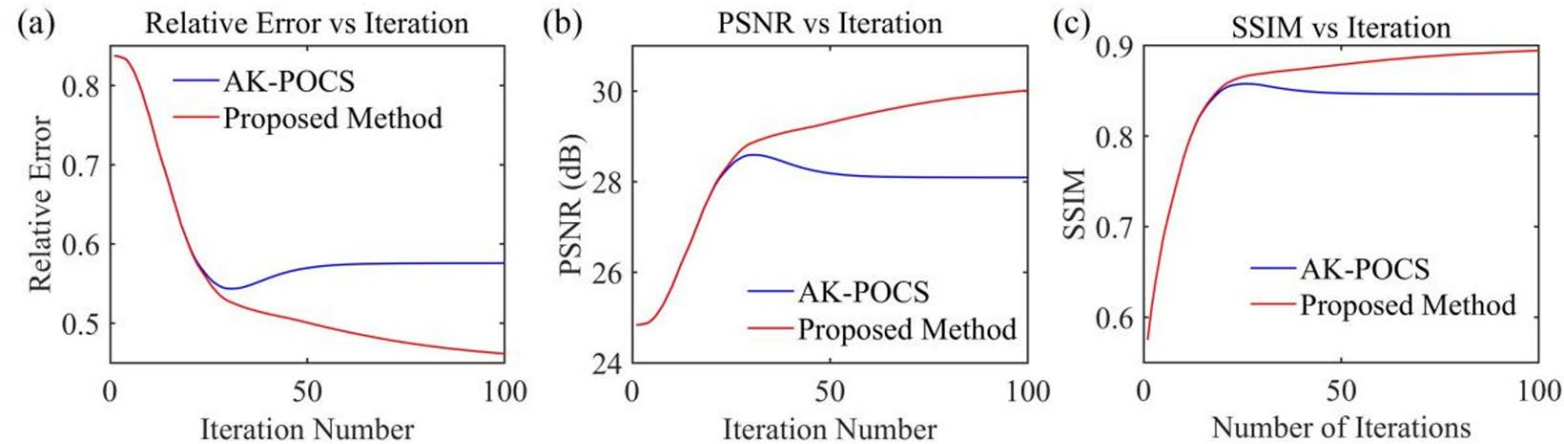
Plots of (**a**) RE, (**b**) PSNR, and (**c**) SSIM versus the number of iterations for post-stack seismic records with 70% missing data.

The subfigures in Fig. 13 show the variation of different indicators with the number of iterations for the AK-POCS method and the proposed method under the condition of 30% data missing. The plots of RE, PSNR, and SSIM demonstrate the superiority of the proposed method. For gentle formations and areas with local structural abrupt changes in field data, the proposed method can not only ensure the continuity of event axes but also has greater advantages over the AK-POCS algorithm in preserving the reflection characteristics of original geological bodies, providing more reliable data support for subsequent structural interpretation. Figures 14, 15, 16 and 17 respectively show the interpolation results and the variation trends of different indicators with the number of iterations under 50% and 70% data missing. Consistent with the prestack interpolation results, the F-K spectrum of the proposed method also has a higher SNR.

## Conclusion

In this study, a new triple convex set projection interpolation method (ALC-POCS method) was constructed by introducing inter-trace correlation lateral constraints, combined with data constraints and sparsity constraints. By integrating a similarity matrix, this method effectively addresses issues such as high interpolation noise and unstable interpolation performance of conventional convex set projection methods. Theoretical derivation and real data experimental verification show that the interpolation results of this method exhibit better event continuity, smaller errors, and stronger consistency between F-K spectra before and after interpolation. It effectively improves the accuracy of seismic data reconstruction and the fidelity of geological features under complex missing patterns, and exhibits particular advantages in processing seismic data with strong lateral correlation, thus providing a high-quality data foundation for subsequent seismic data processing steps.

The distribution characteristics of the correlation matrix of the ALC-POCS method have a strong coupling relationship with the underground geological structure. It shows significant advantages in areas with gentle strata and uniform lithology corresponding to high correlation coefficients, but its performance is comparable to conventional methods in fault zones and lithological interface abrupt change zones corresponding to low correlation coefficients. Meanwhile, although this method outperforms conventional methods in scenarios with consecutive multiple missing traces, the interpolation reliability decreases significantly as the number of consecutive missing traces increases. The core reason is that the correlation matrix still needs optimization under extreme missing scenarios. Future research will optimize the construction logic of the correlation matrix to improve the interpolation performance in low correlation coefficient areas and scenarios with consecutive multiple missing traces.

## Supplementary Information

Below is the link to the electronic supplementary material.

Supplementary Material 1

## Data Availability

The datasets used and/or analyzed during the current study available from the corresponding author on reasonable request. The source codes are available for downloading at the link: https://github.com/ziyu-Qin/ALC-POCS.
