# Supplementary material for: Adaptive lateral constraint-driven POCS interpolation method
Source: Sci Rep. 2026 Mar 1;16:11518. doi: 10.1038/s41598-026-39281-1 (PMC13056980; doi:10.1038/s41598-026-39281-1)
Supplement: Supplementary file 1 — Supplementary Material 1 [file 41598_2026_39281_MOESM1_ESM.docx]

**Appendix A: Convergence Proof of the Algorithm**

***Proposition:***

Let $P_{A}$, $P_{B}$ and $P_{C}$correspond to the data constraint set, frequency-domain sparse set, and lateral constraint set, respectively. When , the iterative sequence $z_{k+1}=P_{A}\left(P_{C}\left(P_{B}\left(z_{k}\right)\right)\right)$ converges to a point in $A\cap B\cap C$.

***Proof of Proposition:***

1. Convex Set Verification

The data constraint set A and the frequency-domain sparse set B have been proven to be convex sets in previous studies. Now, we only need to prove that the lateral constraint set C is a convex set. According to the POCS theory, for a set , if for any $x,y\in N$and any $\lambda\in\left[0,1\right]$, the convex combination $\lambda x+\left(1-\lambda\right)y\in N$, then the set is a convex set^45^. It is known that $x_{1},x_{2}\in C$, and the $l_{1}$-norm is a convex function, and $\left\|x_{1}D\right\|_{1}\leq\gamma$, $\left\|x_{2}D\right\|_{1}\leq\gamma$. For any $\alpha\in\left[0,1\right]$,$\alpha x_{1}+\left(1-\alpha\right)x_{2}$ satisfies:

| $\begin{matrix} \left\Vert\left(\alpha x_{1}+\left(1-\alpha\right)x_{2}\right)D\right\Vert_{1}=\left\Vert\alpha\left(x_{1}D\right)+\left(1-\alpha\right)\left(x_{2}D\right)\right\Vert_{1} \\ \text{ }\leq\alpha\left\Vert x_{1}D\right\Vert_{1}+\left(1-\alpha\right)\left\Vert x_{2}D\right\Vert_{1} \\ \text{ }\leq\alpha\gamma\text{+}\left(1-\alpha\right)\gamma=\gamma\end{matrix}$ | (A.1) |
| --- | --- |

Thus,$\alpha x_{1}+\left(1-\alpha\right)x_{2}\in C$, which means $C$ is a convex set.

1. Nonexpansiveness of Projection Operator
2. $P_{A}$ is nonexpansive

For any，$P_{A}\left(z_{1}\right)-P_{A}\left(z_{2}\right)=\left(I-M\right)\odot\left(z_{1}-z_{2}\right)$.

Therefore,

| $\left\Vert P_{A}\left(z_{1}\right)-P_{A}\left(z_{2}\right)\right\Vert_{F}=\left\Vert\left(I-M\right)\odot\left(z_{1}-z_{2}\right)\right\Vert_{F}\leq\left\Vert z_{1}-z_{2}\right\Vert_{F}$ | (A.2) |
| --- | --- |

1. $P_{B}$ is nonexpansive

The Fourier transform is a unitary transform, satisfying $\left\|\Gamma\left(z\right)\right\|_{F}=\left\|z\right\|_{F}$, The hard-threshold projection satisfies:

| $\begin{matrix} \left\Vert P_{B}\left(z_{1}\right)-P_{B}\left(z_{2}\right)\right\Vert_{F}=\left\Vert\Gamma^{-1}\left(\Gamma\left(z_{1}\right)\odot\chi_{1}-\Gamma\left(z_{2}\right)\odot\chi_{2}\right)\right\Vert_{F} \\ \text{ }=\left\Vert\Gamma\left(z_{1}\right)\odot\chi_{1}-\Gamma\left(z_{2}\right)\odot\chi_{2}\right\Vert_{F} \\ \text{ }\leq\left\Vert\Gamma\left(z_{1}\right)-\Gamma\left(z_{2}\right)\right\Vert_{F}=\left\Vert z_{1}-z_{2}\right\Vert_{F} \end{matrix}$ | (A.3) |
| --- | --- |

1. $P_{C}$ is nonexpansive

$P_{C}$is a proximal projection under $l_{1}$-norm constraint. According to convex optimization theory, the $P_{C}$ satisfies nonexpansiveness.

In summary, $P_{A}\left(P_{C}\left(P_{B}\left(z_{k}\right)\right)\right)$ is a nonexpansive operator.

1. Non-emptiness of Intersection

In the actual seismic data interpolation process, data consistency and frequency-domain sparsity are satisfied, and the spatial continuity of seismic wave propagation ensures similarity between adjacent traces. Therefore,.

By the classic POCS convergence theorem, let $\left\{S_{1},S_{2},\cdots,S_{N}\right\}$ be convex sets in , and . Let $P_{S_{i}}$be the nonexpansive projection operator of $S_{i}$. Then, the iterative sequence $z_{k+1}=P_{S_{1}}\left(P_{S_{2}}\left(\ldots P_{S_{N}}\left(z_{k}\right)\ldots\right)\right)$converges to a point in $\cap_{i=1}^{N}S_{i}$. In the ALC-POCS algorithm:

The convex sets $A,B,C$ satisfy (proven);

The projection operators $P_{A},P_{B}\text{,}P_{C}$ are all nonexpansive (proven);

The iterative sequence $\left\{z_{k}\right\}$is in the finite-dimensional space . The sequence generated by the iteration of nonexpansive operators is a Cauchy sequence, so there exists a limit $z_{\infty}=\lim_{k\to\infty}z_{k}$.Taking the limit of the iterative formula, $z_{\infty}=P_{A}\left(P_{C}\left(P_{B}\left(z_{\infty}\right)\right)\right)$, i.e., $z_{\infty}=A\cap B\cap C$. Therefore, the iterative sequence of the ALC-POCS algorithm converges to the optimal interpolation solution in $A\cap B\cap C$.
